# Diabetes Telemedicine Mediterranean Diet (DiaTeleMed) Study: study protocol for a fully remote randomized clinical trial evaluating personalized dietary management in individuals with type 2 diabetes

**DOI:** 10.21203/rs.3.rs-4492352/v1

**Published:** 2024-06-25

**Authors:** Lauren T. Berube, Collin J. Popp, Margaret Curran, Lu Hu, Mary Lou Pompeii, Souptik Barua, Emma Bernstein, Vanessa Salcedo, Huilin Li, David E. St-Jules, Eran Segal, Michael Bergman, Natasha J. Williams, Mary Ann Sevick

**Affiliations:** New York University Grossman School of Medicine; New York University Grossman School of Medicine; New York University Grossman School of Medicine; New York University Grossman School of Medicine; New York University Grossman School of Medicine; New York University Grossman School of Medicine; New York University Grossman School of Medicine; New York University Grossman School of Medicine; New York University Grossman School of Medicine; University of Nevada Reno; Weizmann Institute of Science; New York University Grossman School of Medicine; New York University Grossman School of Medicine; New York University Grossman School of Medicine

**Keywords:** precision nutrition, glycemic variability, continuous glucose monitors, dysglycemia, remote patient monitoring, randomized clinical trial

## Abstract

**Background:**

The Diabetes Telemedicine Mediterranean Diet (DiaTeleMed) Study is a fully remote randomized clinical trial evaluating personalized dietary management in individuals with type 2 diabetes (T2D). The study aims to test the efficacy of a personalized behavioral approach for dietary management of moderately-controlled T2D, versus a standardized behavioral intervention that uses one-size-fits-all dietary recommendations, versus a usual care control (UCC). The primary outcome will compare the impact of each intervention on the mean amplitude of glycemic excursions (MAGE).

**Methods:**

Eligible participants are between 21 to 80 years of age diagnosed with moderately-controlled T2D (HbA1c: 6.0–8.0%), and managed on lifestyle alone or lifestyle plus metformin. Participants must be willing and able to attend virtual counseling sessions and log meals into a dietary tracking smartphone application (DayTwo), and wear a continuous glucose monitor (CGM) for up to 12 days. Participants are randomized with equal allocation (n = 255, n = 85 per arm) to one of three arms: 1) *Personalized*, 2) *Standardized*, or 3) *UCC*. Measurements occur at 0 (baseline), 3, and 6 months. All participants receive isocaloric energy and macronutrients targets to meet Mediterranean diet guidelines plus 14 intervention contacts over 6 months (4 weekly then 10 biweekly) to cover diabetes self-management education. The first 4 *UCC* intervention contacts are delivered via synchronous videoconferences followed by educational video links. Participants in *Standardized* receive the same education content as *UCC* on the same schedule. However, all intervention contacts are conducted via synchronous videoconferences, paired with Social Cognitive Theory (SCT)-based behavioral counseling, plus dietary self-monitoring of planned meals using a mobile app that provides real-time feedback on calories and macronutrients. Participants in the *Personalized* arm receive all elements of the *Standardized* intervention, plus real-time feedback on predicted post-prandial glycemic response (PPGR) to meals and snacks logged into the mobile app.

**Discussion:**

The DiaTeleMed study will address an important gap in the current landscape of precision nutrition by determining the contributions of behavioral counseling and personalized nutrition recommendations on glycemic control in individuals with T2D. The fully remote methodology of the study allows for scalability and innovative delivery of personalized dietary recommendations at a population level.

**Trial registration:**

The DiaTeleMed Study is registered with ClinicalTrials.gov (Identifier: NCT05046886)

## Background and rationale

Type 2 diabetes (T2D) affects 37.3 million people in the United States (U.S.) (1). T2D is a chronic, progressive condition that can lead to long-term cardiovascular consequences, such as heart and kidney disease, stroke, retinopathy, and amputation (2). Early management of T2D is critical to prevent complications, as studies suggest that vascular risks developing during periods of poor glycemic control in the initial stages of T2D are only partially remedied by subsequent, better glycemic management (3–5).

As postprandial glycemic excursions are major determinants of glycemic control in early T2D (6, 7), dietary management is key to successful treatment. Dietary recommendations for T2D aim to minimize postprandial glycemic response (PPGR) (8); however, there is limited evidence regarding the best dietary approach to minimize PPGR. Current strategies are based on one-size-fits-all dietary recommendations (e.g., low glycemic index or low carbohydrate), but the results of clinical intervention studies do not show these strategies to be unequivocally more efficacious than other diets for glycemic control (9–17). One-size-fits-all approaches may fail to manage glycemia for individuals with T2D because they do not consider the interindividual variability in PPGR to the same foods (18–20), which is influenced by characteristics such as lifestyle, metabolism, and the composition and function of the gut microbiome (21, 22). Consequently, individuals with T2D who follow one-size-fits-all approaches may experience postprandial glycemic excursions despite their best efforts to adhere to recommended diets.

The Personal Nutrition Project (PNP) constructed the first personalized machine learning algorithm for predicting PPGR (hereafter, PNP algorithm) (18). Personalizing dietary recommendations to an individual’s unique PPGR using the PNP algorithm is a proactive approach to dietary management for patients with moderately-controlled T2D that could increase mastery and self-management success beyond what can be achieved through a one-size-fits-all diet. Studies demonstrate that the PNP algorithm is more predictive of PPGR than nutrition content alone (18, 19, 23). In a randomized clinical trial (RCT) comparing the clinical efficacy of a 6-month Mediterranean diet to a 6-month PNP algorithm-guided diet in 225 Israeli adults with prediabetes, participants randomized to the PNP algorithm-guided diet had greater reductions in continuous glucose monitoring (CGM)-based measures, such as daily time above 140 mg/dL and mean glucose, and metabolic parameters, such as HbA1c and triglycerides (24). Similarly, in a preliminary 2-week randomized crossover trial in 23 Israeli adults with newly diagnosed T2D, the PNP algorithm-guided diet resulted in significantly lower levels of glycemic exposure than the Mediterranean diet (25). Following the crossover trial, 16 participants completed an additional 6 months of the PNP algorithm-guided diet, with significant improvements in multiple metabolic parameters. Of 13 participants who started the intervention with HbA1c ≥ 6.5%, 61% achieved HbA1c < 6.5% at 6 months (25). These findings are promising for individuals with T2D; however, both studies were conducted in Israel, which limits generalizability to a U.S. population. The Israeli trial in patients with T2D was a pilot study, and the 6-month follow-up component did not include a control group. Thus, larger scale randomized clinical trials are needed in U.S. adults with T2D to validate the clinical efficacy of the PNP algorithm-guided diet.

The DiaTeleMed Study is a fully remote RCT that tests the efficacy of the PNP algorithm to reduce glycemic exposure in U.S. adults with moderately controlled T2D. Using a three-arm design that includes a usual care control (*UCC*), a standardized behavioral intervention that uses one-size-fits-all dietary recommendations (*Standardized*), and a personalized behavioral approach leveraging the PNP algorithm (*Personalized*), we will determine the incremental contributions of behavioral counseling and precision nutrition recommendations to glycemic control. Here, we describe the study protocol.

## Objectives

The purpose of the DiaTeleMed Study is to determine the efficacy of *Personalized* compared to *Standardized* and *UCC*. Although hemoglobin A1c (HbA1c) has traditionally been measured in clinical interventions for T2D to assess glycemic control (26–28), there is growing evidence that glycemic variability (GV), defined by postprandial glycemic excursions and hypoglycemic nadirs, may be a better treatment target (29–31). Thus, the primary outcome will compare the impact of each intervention on GV, measured as the mean amplitude of glycemic excursions (MAGE). Our primary hypothesis is MAGE_Personalized_< MAGE_Standardized_< MAGE_UCC_ at 6 months. We will measure HbA1c as a secondary outcome to allow comparability with prior research. Exploratory outcomes are changes in β-cell function, the medication regimen, and other measures of GV. We will also explore the mediation effect of self-efficacy on the relationship between randomization assignment and GV.

## Trial Design

**Figure 1:**
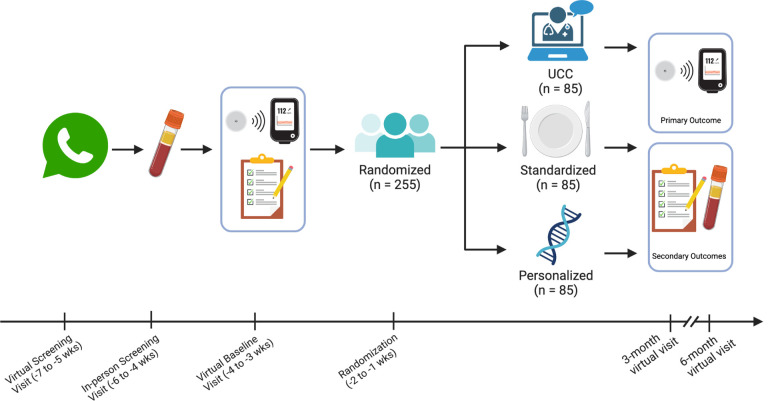
Overview of study design and measurement timepoints Patients who are interested in enrolling in the study complete a virtual screening to determine eligibility. Eligible participants are scheduled for an in-person blood draw at a Quest Laboratory location of their choice to confirm HbA1c eligibility requirements before participating in study activities. Prior to measurement timepoints, participants are mailed CGM sensors and a CGM reader. At each measurement timepoint, participants complete electronic questionnaires, wear a CGM sensor for 12 days, and visit a Quest Laboratory location for an in-person blood draw. After the baseline assessment, participants are randomized to one of three study intervention arms: *UCC*, *Standardized*, or *Personalized*.Additional measurements occur at 3 and 6 months.

The DiaTeleMed Study is a three-arm RCT in adults with moderately-controlled T2D. Participants are randomized with equal allocation (n = 255, n = 85 per arm) to one of three arms: 1) *Personalized*, 2) *Standardized*, or 3) *UCC*. Measurements occur at 0 (baseline), 3, and 6 months. This protocol was prepared using the Standard Protocol Items: Recommendations for Intervention Trials (SPIRIT) reporting guidelines (see SPIRIT checklist in Additional File 1) (32). Figure 1 and Table 1 provide an overview of the study design and study schedule, respectively.

## Methods

### Participants, interventions, and outcomes Study Setting

The study protocol, including the intervention and all measurements, is conducted using entirely remote methods by study staff at New York University Langone Health (NYULH) in New York, NY.

### Eligibility

To be eligible for this study, participants are between 21 to 80 years of age with well-to-moderately controlled T2D, and willing and able to attend virtual counseling sessions and log meals into a dietary tracking smartphone application. Participants are also free of antibiotic or antifungal therapy for at least 3 months prior to enrollment. Additionally, participants complete a 7-day run-in during which they log at least 2 meals per day into the dietary tracking smartphone application. In the initial stages of the study, well-to-moderately controlled T2D was defined as an HbA1c 6.5–8.0% controlled by diet-alone or diet plus metformin management. However, due to lags in recruitment, the lower limit of HbA1c was reduced to 6.0%, and we dropped the 7-day run-in following the observation that few participants were excluded for nonadherence to self-monitoring (n = 10, 6%). T2D medication regimens other than metformin are exclusionary because of their substantive effects on PPGR. Eligibility criteria are listed in Table 2.

### Recruitment, screening, and enrollment procedures

#### Recruitment

We leverage the NYULH electronic health record (EHR) capabilities to recruit participants who receive care at NYULH affiliated practices. Using the EHR, we develop a list of potentially eligible patients using the International Classification of Diseases (ICD)-10 codes for the diagnosis of T2D and/or HbA1c 6.0–8.0%. Potentially eligible patients are sent a message through the patient portal. Those who are interested self-refer by selecting a link that automatically notifies the study staff, who contact patients directly for screening and enrollment. Recruitment began in October 2021 and is ongoing.

#### Screening and enrollment

Patients who self-refer are contacted by telephone for pre-screening to determine eligibility. Eligible participants are scheduled for a virtual screening assessment via WebEx (Cisco, San Jose, CA, USA), a HIPAA compliant videoconferencing platform. During the virtual screening, participants sign an electronic informed consent via Research Electronic Data Capture (REDCap) software (Vanderbilt University, Nashville, TN, USA). Study staff assist participants with loading the following mobile apps to their smartphone device: the DayTwo Personalized Nutrition Recommendations dietary tracking app (DayTwo Inc., Tel Aviv-Yafo, Israel) and the WebEx videoconferencing app. Those requiring a smartphone are provided loaner phones and no-cost service plans to use for the duration of the study. Participants are virtually trained by study staff to use the DayTwo app to self-monitor their daily diet, including how to search for food items and save favorite mixed meals. All participants are scheduled for venipuncture by a certified phlebotomist at a Quest Laboratory (Quest Diagnostics, Secaucus, NJ) location of their choice to confirm that HbA1c meets eligibility requirements, and a subset of participants have fasting insulin and glucose measured. To remain eligible for the study, participants must meet HbA1c eligibility.

### Measurements

Measurements are conducted at 0 (baseline), 3, and 6 months via Webex. REDCap is used for all data collection and management procedures. In advance of the baseline measurement visit, a bathroom scale is mailed to participants who do not own one. Prior to each measurement timepoint, participants are mailed Abbott FreeStyle Libre Pro CGM sensors and a CGM reader (Abbott Park, IL), which are returned to the investigators in a prepaid postage box. Participants are sent a YouTube video on CGM insertion prior to their scheduled Webex meeting, during which they are guided by study staff to self-insert the CGM sensor. To prevent detachment of the CGM device, the skin surface is prepared with SkinTac (TORBOTGroup, Inc., Cranston, RI) and, once inserted, covered with a Simpatch (Triad Co., Ltd, Seoul, South Korea) adhesive patch. At each measurement timepoint, participants are instructed to wear the CGM sensor for a period of 12 days. Monetary incentives are provided at each measurement timepoint.

### Primary outcome

#### MAGE.

MAGE, the most commonly reported measure of GV, assesses the variation about the mean by summing the absolute rises or falls of daily glucose levels, ignoring excursions < 1 standard deviation (SD). MAGE will be evaluated via the CGM, which captures blinded interstitial glucose readings every 15 minutes for up to 2 weeks from the sensor applied to the participant’s upper arm. Participants use the CGM Reader to initiate the sensors and confirm that they are functioning properly. After confirming proper function, participants are directed to place the reader into an opaque mail packet and seal it. This process ensures that participants remain blinded to CGM sensor readings during the measurement period. Participants who are taking aspirin or vitamin C are asked to discontinue their use during the measurement period as they can influence CGM accuracy. The CGM sensors are worn for 12 days and are returned after the wear time in a prepaid sharps box. To calculate MAGE from CGM sensor data, we will use EasyGV 9.0.R2 software (Nathan R. Hill, University of Oxford, United Kingdom) (33).

### Secondary outcome

#### HbA1c.

Since most studies conducted in individuals with T2D are evaluated using HbA1c, we will evaluate between-group differences in HbA1c changes to permit comparison with existing literature. Glycosylated hemoglobin is evaluated from blood sampling (~ 10 ml) obtained via venipuncture by a certified phlebotomist at Quest Diagnostics and evaluated in the Quest Clinical Laboratory Improvement Amendments of 1988 (CLIA)-certified lab.

### Exploratory outcomes

#### β-cell function.

In a subset of participants, fasting insulin and glucose are obtained via venipuncture (~ 15ml) at Quest Diagnostics and evaluated in the Quest CLIA-certified lab. We will use the homeostatic model assessment (HOMA2 model) (34).

#### Changes in the medication regimen.

At baseline, participants provide a list all prescribed and over-the-counter medications. At 3 and 6 months they are asked to report initiation or discontinuation of diabetes and weight loss medications since the prior measurement visit.

#### Non-MAGE GV measures.

CGM data captured from the Abbott FreeStyle Libre Pro sensor will also be used to generate non-MAGE measures of GV, including coefficient of variation (CV), continuous overall net glycemic action (CONGA), mean area under the curve (AUC) of the blood glucose levels following meals, number of events and total time during the week in which glucose levels > 140 mg/dL, and time-in-range (70–180 mg/dL) (35–37). We will investigate measures of GV overall and stratified by daytime and nighttime glycemia based on recent CGM guidelines and evidence (36, 38). Analyzing other measures of GV will allow our results to be compared to other literature and will enhance interpretability of findings. All values will be calculated using EasyGV 9.0.R2 software (33).

### Mediator

#### Self-efficacy.

Self-efficacy for diabetes management will be assessed using the well-validated, 8-item Stanford Diabetes Self-Efficacy Scale (39). Participants are asked to rate their confidence in completing various activities related to self-management of T2D on a scale ranging from 1 (not at all confident) to 10 (totally confident). An overall score will be computed by summing items, and this score will be used to evaluate the mediating effect of self-efficacy on the relationship between randomization assignment and GV.

### Covariates

#### Sociodemographic characteristics, habits, and health history.

At baseline, REDCap is used to collect sociodemographic characteristics, habits, and clinical history, including: age, race, sex, comorbid conditions, living arrangement, education, employment, income, health insurance, country of origin for self. At each measurement timepoint, participants self-report any changes to the medication regime.

#### Anthropometrics, physical activity and sleep.

At each measurement timepoint, participants self-report their weight using their home scale or a scale sent to them by the study staff. Height is also self-reported at baseline. Body mass index (BMI) is calculated from self-reported weight and height.

#### Dietary intake.

At each measurement timepoint, 24-hour dietary recalls are collected using the Automated Self-Administered 24-Hour (ASA24) dietary assessment tool (National Cancer Institute, Bethesda, MD). Participants report dietary intake from midnight to midnight of the previous day. Using the USDA’s Food and Nutrient Database for Dietary Studies, food and beverage items reported are automatically converted to energy and nutrient values (including macronutrients, vitamins, minerals, carotenoids, fats and cholesterol, specific fatty acids, and other substances) and food categories (e.g., fruits, grains, protein, fats, vegetables, dairy, added sugar, alcohol). In the beginning of the study, 3 unannounced ASA24 recalls were collected, including two weekdays and one weekend day. At the study midpoint, to reduce participant burden, recalls were reduced to a single unannounced measurement at each time point.

### Study Interventions

The study design permits evaluation of the incremental benefits of behavioral counseling and personalization of diet beyond what can be achieved with routine diabetes education, while holding constant the frequency of intervention contacts between the 3 arms (weekly for the first 4 weeks and then every other week for 20 weeks). All live group sessions are conducted by registered dietitian nutritionists who are certified diabetes educators. Sessions are anchored by brief animated videos, interspersed with scripted, open-ended questions and exercises designed to guide discussion, resulting in a videoconferenced sessions lasting 60 minutes.

### All Study Arms

**Figure 2:**
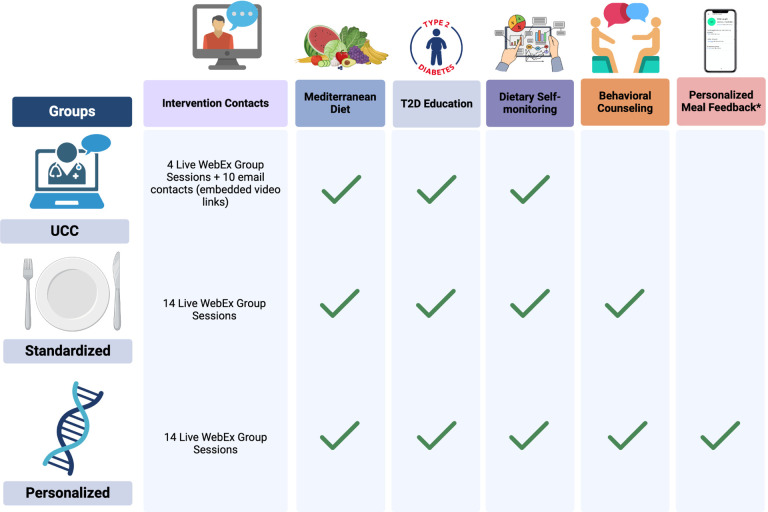
Intervention components by study arm Participants in all arms receive 4 live Webex group sessions, which provide education on T2D management and isocaloric Mediterranean diet recommendations. All participants have access to the DayTwo mobile app to self-monitor dietary intake and receive real-time feedback on dietary composition of meals. After the 4 live sessions, participants in the *UCC*arm are sent 10 emails with links to educational videos, while participants in the *Standardized* and *Personalized* arms participate in 10 live Webex behavioral counseling group sessions. Participants in the *Personalized*arm receive real-time, personalized meal feedback on predicted PPGR from the DayTwo app.

To ensure that participants are operating with the same basic knowledge of T2D management, the study begins with 4 weekly sessions featuring diabetes self-management education, delivered via WebEx videoconferencing. All participants are provided with a nutrition prescription that includes an isocaloric energy target (calculated with the Mifflin St Jeor Eq. (40) and adjusted for self-reported physical activity level from the baseline questionnaire) and macronutrient targets to meet Mediterranean diet recommendations (50% of calories from carbohydrates, 30% from fat, < 10% from saturated fat, and 20% from protein; see **Supplemental File**). All participants are provided with education regarding the Mediterranean diet, which has been recommended for dietary management of T2D by the American Diabetes Association (41). The Mediterranean diet guidelines encourage consumption of a primarily plant-based diet, minimally processed foods (e.g., fruits and vegetables, whole grains, beans and peas, nuts and seeds) with high quality fats, low to moderate amounts of fish, eggs, poultry, and dairy, and limited consumption of sweets and fatty or processed meats. Figure 2 shows the intervention components for each group.

#### Usual Care Control Arm

Following the initial 4 sessions, UCC participants receive 10 biweekly emails containing links to additional educational videos. Participants in the *UCC* arm have access to the DayTwo app to monitor their diet and obtain real time feedback on calories and macronutrients. After week 4, they are advised that, if desired, they may discontinue use of the app.

#### Standardized Arm

**Figure 3:**
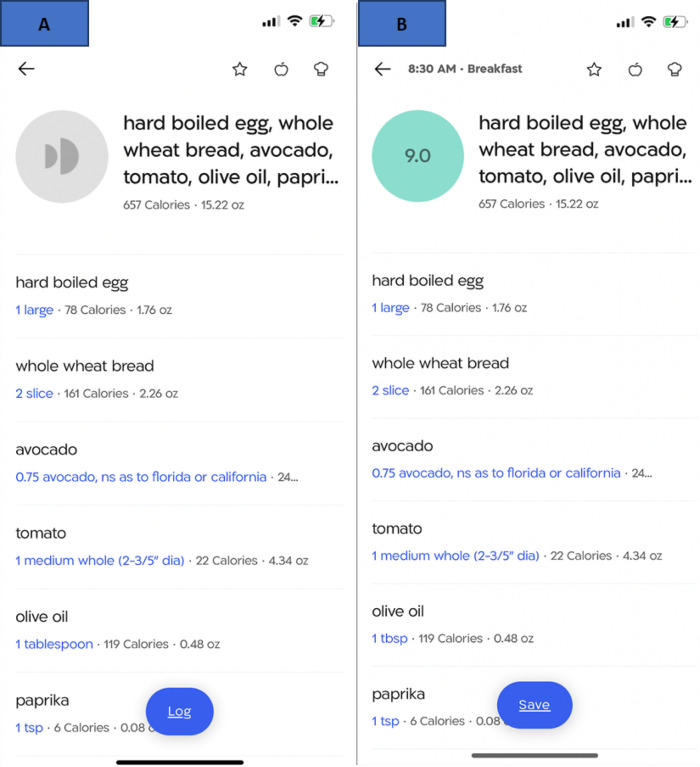
Screenshots of the feedback provided by the DayTwo app Panel A displays the feedback that the *Standardized*arm receives after entering a meal, while panel B displays the feedback that the *Personalized* arm receives, including the color-coded PPGR meal score. A higher score indicates a better PPGR score.

Following the initial 4 sessions, *Standardized* participants attend 10 biweekly live group sessions. The content of the *Standardized* intervention includes the same educational content delivered to the UCC arm via video links, plus behavioral counseling based in Social Cognitive Theory (SCT) (42, 43), with an emphasis on building self-efficacy to engage in healthy behaviors to manage T2D. Participants in the *Standardized* arm use the DayTwo mobile app throughout the intervention to log meals in advance of consuming them, and use real-time graphical and numerical feedback from the app on the accumulation of total calories and grams of carbohydrates, fats, and protein (Fig. 3) to guide dietary decisions with particular emphasis on carbohydrates.

#### Personalized Arm

Personalized participants receive all of the elements of the *Standardized* intervention. *Personalized* participants use the DayTwo app to log meals in advance of consuming them, and receive the same feedback from the app as the *Standardized* participants plus real-time feedback on their predicted PPGR to planned meals and snacks, generated using the PNP algorithm described in more detail elsewhere (18). In brief, participants collect a stool sample using the OMNIgene-GUT stool collection kit (OMNIgene-GUT; DNA Genotek, Ottawa, ON, Canada) and ship their sample directly to DayTwo in a prepaid mail package. Sex, date of birth, height, weight, physical activity, HbA1c, and CGM data are assembled by the NYULH team on a secure cloud-based server accessible to the DayTwo team^1^. The DayTwo team uses the PNP algorithm to generate PPGR scores that are displayed when participants in the *Personalized* arm enter a planned meal into the app. PPGR scores vary from 1 to 10, with 1 being the worst possible PPGR and 10 being the best. PPGR scores are also color-coded per a traffic light motif, with “green” scores (score of 8 to 10) indicating an acceptable PPGR for that meal (see Fig. 3). In a counseling session called “Getting to Green” (see Table 3), participants in the *Personalized* arm are trained to modify their food choices when they receive PPGR scores coded “yellow” (score of 6 to 7.9) or “red” (score of 1 to 5.9). They are advised to review the ingredients of their planned meal and experiment with food substitutions, portions, and/or add limited amounts of a healthy fat to slow the absorption of carbohydrates, all while maintaining their calorie target for the meal.

### Statistical Methods

#### Sample size.

This study is powered to test the hypothesis that MAGE_*Personalized*_< MAGE_*Standardized*_ < MAGE_*UCC*_ at 6 months. In the pilot cross-over study in patients with T2D described earlier (25), Segal et al. found that, during 2 weeks of exposure to the PNP-guided diet, MAGE was 14.1 mg/dL lower than that observed during 2 weeks of exposure to a Mediterranean diet. Following this crossover component of this pilot, participants were then directed to follow the PNP-guided diet for an additional 6 months, demonstrating a reduction in MAGE of 26.7mg/dL ± 16.3 (p < 0.001). This change was associated with multiple, clinically significant, metabolic improvements. While we expect larger between-group differences to be achievable, we conservatively powered the study to detect the smaller MAGE difference of 14.1 mg/dL observed by Segal et al (25). Tuncan recently reported that MAGE SDs in patients with T2D range from 15 to 25 mg/dL (44). We conservatively assumed a SD of 25 mg/dL and, based on a two-tailed t-test and an alpha of 0.0167 (to account for 3 multiple comparisons), a final sample of 204 (68/arm) is required to detect a difference in means of 14.1 mg/dL with a power of 80%. With 204 participants, we can also detect a reduction in HbA1c as small at 0.56% with 80% power, based on a two-sample two-tailed t-test, an alpha of 0.0167, and assuming SD of 1.00% (derived from our prior studies). To account for expected drop-out of 20%, we aim to enroll 255 participants.

#### Randomization and blinding.

Allocation sequence is generated using computer-generated random numbers by the study biostatistician who is not involved in enrollment and intervention delivery. Due to the nature of the behavioral intervention, the participants and the dietitian/interventionist are not blinded to the intervention. The biostatistician will be blinded to arm assignments during data analysis. Additionally, participants are blinded to their CGM readings during the study.

#### Statistical analysis.

We will use an intent-to-treat approach to analyze primary and secondary outcomes. All participants will be included in the data analysis in the treatment arm to which they were randomized, regardless of compliance, treatment received, or deviation from the protocol. Data from participants who withdraw will be used to the extent permitted by human subjects and privacy considerations.

Primary and secondary analyses will be conducted using linear mixed models (for continuous outcomes), logistic generalized linear mixed models (for binary outcomes), and random effects multinomial models (for outcomes with more than 2 levels) to determine intervention effects on longitudinal variables at 0, 3, and 6 months. In all models, time and intervention will be included as fixed effects, and participant will be the random effect. The intervention effect of interest is the treatment×time interaction in this model. Covariates, including identified predictors of missing data and sociodemographic and clinical covariates will be included as necessary in adjusted analyses. The primary and secondary analyses will be done using STATA version 15.1 software (StataCorps LLC, College Station, TX, USA) and Statistical Analysis Software version 9.4 (SAS Institute Inc., Cary, NC, USA).

### Adherence

We will evaluate adherence to the intervention in terms of: 1) the percent of scheduled counseling sessions attended (all arms during first 4 weeks, *Personalized* and *Standardized* arms during weeks 5–24); 2) the number and percent of days during which participants logged at least one meal into the DayTwo app (all arms); and 3) intermediate changes in diet (calories and macronutrient distribution) based on ASA24 dietary recalls (all arms).

### Safety

During the study, participants’ clinical laboratory values (e.g., HbA1c) are monitored. All laboratory results appear in NYULH EHR system and are visible to the research physician (MB), who will take corrective action or contact the physician of record if abnormal values are obtained. Participants are reminded that this intervention is not a replacement for usual care and are instructed to attend their usual care visits with their physician during the study.

## Discussion

Current dietary interventions for individuals with T2D to manage PPGR are based on one-size-fits-all dietary recommendations that do not consider the interindividual variability in glycemic response to foods. Advances in precision nutrition have elucidated the multitude of factors that influence individual response to diet and have allowed for development of innovative nutrition algorithms to predict physiological response to foods. The PNP algorithm (18), which considers individual factors that influence PPGR, such as the gut microbiome, provides targeted, actionable dietary recommendations that may help individuals with T2D make optimal dietary decisions to improve glycemic control. To our knowledge, this is the first fully-powered study to test the efficacy of a personalized behavioral intervention on dietary management for individuals with moderately-controlled T2D.

The DiaTeleMed study has several strengths that will add to the current literature. First, we are comparing a personalized diet to an optimal generic Mediterranean diet, which serves as a strong benchmark against which the precision nutrition intervention will be compared. As such, we can elucidate any observed differences in outcomes associated with the personalized intervention. Second, the three-arm study design allows for multiple intervention comparisons; thus, allowing for a comprehensive assessment of standard of care. Finally, the randomized nature of a clinical trial minimizes bias and confounding factors, thus enhancing the internal validity of the study.

Despite the inherent strengths, there are limitations to the current study. First, some participants (e.g., those who do not want to change behavior or are reticent to use technology) may not elect to participate in the study, which could limit generalizability to the most compliant and technology-savvy individuals. Second, we are conducting the study within one urban healthcare system, NYULH, which limits generalizability to other healthcare systems, particularly those in rural settings. Third, as the intervention involves self-monitoring using software available in English, non-English speaking individuals are excluded from participating. However, if efficacious, the Personalized intervention can be adapted for non-English speaking individuals and tested in subsequent trials. Fourth, due to the nature of behavioral interventions, participants and interventionists are not blinded to randomization arm. Finally, participants who are not randomized to the *Personalized* arm may be disappointed by randomization assignment and less engaged in the intervention.

The NIH Common Fund expects precision nutrition to become a mainstay in medical care by 2030 (21). Personalized nutrition leverages biological, behavioral, social, and environmental data to make more precise and effective dietary recommendations. However, delivering personalized dietary recommendations at a population level will require innovative and scalable methodologies, such as the fully remote methodology described here, which was informed by our recently completed Personal Diet Study (45). The DiaTeleMed study will address an important gap in the current landscape of precision nutrition by determining the contributions of behavioral counseling and personalized nutrition recommendations on glycemic control in individuals with T2D. The fully remote methodology of the study allows for scalability and innovative delivery of personalized dietary recommendations at a population level.

### Trial Status

The study protocol is based on version date July 7, 2020. Study recruitment started October 2021 and recruitment is currently ongoing. It is anticipated that recruitment will be completed in November 2025.

## Figures and Tables

**Table 1:** Schedule for enrollment, interventions and assessments (SPIRIT figure)

	STUDY PERIOD					
	Enrollment	Baseline assessment	Allocation	Post-allocation	
	−7 to −4 weeks	−4 to −3 weeks	−2 to −1 weeks	3 months	6 months
**Enrollment:**					
Virtual eligibility screen	X				
Informed consent	X				
HbA1c eligibility screen (venipuncture)	X				
**Allocation**			X		
**Interventions:**					
UCC			*------	-------------	------*
Standardized			*------	-------------	------*
Personalized			*------	-------------	------*
**Assessments:**					
*Baseline variables*		X			
Sociodemographic variables					
Habits					
Health history					
Height					
*Primary outcome*		X		X	X
MAGE					
*Secondary outcome*		X		X	X
HbA1c					
*Exploratory outcomes*		X		X	X
β-cell function					
Changes in the medication regimen					
Non-MAGE GV measures (CV, CONGA, Mean AUC following meals, time > 140 mg/dL)					
*Other data variables*		X		X	X
Self-efficacy					
Dietary intake					
Weight					

HbA1c: Hemoglobin A1c; MAGE: Mean amplitude of glycemic excursions; GV: Glycemic variability; CV: Coefficient of variation; CONGA: Continuous Overall Net Glycemic Action; AUC: Area under the curve

**Table 2:** Eligibility criteria for the DiaTeleMed Study

**Inclusion criteria**
21 to 80 years of age Diagnosed with moderately-controlled T2D (HbA1c: 6.0% – 8.0%) Willing and able to use a smartphone to self-monitor diet Willing and able to attend virtual counseling sessions Willing and able to wear a CGM on their arm for 12 days Free of antibiotic or antifungal therapy for at least 3 months prior to enrollment
**Exclusion criteria**
Unable to self-monitor diet using an English-only mobile app (e.g., uncorrected sight impairment, illiterate, non- English-speaking, dementia) Baseline use of T2D medications other than metformin Baseline use of medications for weight loss Chronic use of steroids or immunosuppressants Baseline use of atypical antipsychotics Undergoing chemotherapy Pregnant, planning to become pregnant during the study period, or become pregnant during the study Chronic disease that affects energy/glucose metabolism (e.g., Cushing’s syndrome, acromegaly, hyperthyroidism) Requires special dietary management (e.g., late-stage kidney disease, cirrhosis, HIV) Limited control over diet (e.g., are homeless or institutionalized, in a nursing home or personal care facility, or incarcerated) Bariatric surgery or are unwilling to delay bariatric surgery for the next 7 months Diagnosed with a chronic active inflammatory or neoplastic disease in the past 3 years Diagnosed with a chronic gastrointestinal disorder (e.g. inflammatory bowel disease or celiac disease) Active substance use disorder

T2D: type 2 diabetes; CGM, continuous glucose monitor; HbA1c: glycated hemoglobin

**Table 3:** Intervention content for the DiaTeleMed Study by randomization arm

Week	Video Topic		Randomization assignment		
	Educational content	Behavioral counseling content	UCC	S	P
1	Introduction to T2D. Getting the most out of medical care		x	x	x
		Goals for life. Establishing relevance of behavior change.		x	x
2	Glucose self-monitoring,		x	x	x
	Making sense of blood sugars Dealing with out-of-range blood sugars	Establishing baseline behavior. Setting long-terms goals for glycemic control.		x	x
3	Introduction to the isocaloric Mediterranean die Carbohydrates, Fat, Protein, the Mediterranean Diet & Plate Method		x	x	x
		Using the DayTwo app to establish dietary patterns and set short- term goals related to macronutrient targets.		x	x
4	Physical activity and blood sugar control: Moderate intensity physical activity		x	x	x
		Setting goals		x	x
6	Adding color to your diet		x_v_	x	x
		Turning goals into habits with self-reward		x	x
	“Getting to Green”: Using the DayTwo app to establish dietary patterns and set short- term goals related to minimizing PPGR				x
8	Sources of protein		x_v_	x	x
		Introduction to problem solving		x	x
10	Stress and blood sugar control		x_v_	x	x
		Problem solving: Emotional eating		x	x
12	Physical activity and blood sugar control: resistance training to build muscle mass		x_v_	x	x
		Problem solving: Eliminating self-talk		x	x
14	Limiting meats and sweets		x_v_	x	x
		Problem solving: Food cravings, food addictions		x	x
16	Dealing with dairy		x_v_	x	x
		Problem solving: Anticipating high risk situations		x	x
18	The role of stress in blood sugar control		x_v_	x	x
		Problem solving: Managing stress		x	x
20	Flavoring food without salt		x_v_	x	x
		Problem solving: Lapses and relapses		x	x
22	Medication management in T2D		x_v_	x	x
		Problem solving: Leveraging your medical support team		x	x
24	Communicating with your health care provider about T2D management, now and in the future		x_v_	x	x
		Putting it all together		x	x

UCC: *Usual Care Control arm*; S: *Standardized arm*; P: *Personalized arm*

X_v_ indicates that this arm receives links to educational videos

## Data Availability

The data supporting this study’s findings are available from the corresponding author upon reasonable request.

## Footnotes

1. To protect privacy, data shared with DayTwo via the cloud are identified by a study ID number that cannot be linked by DayTwo with their identity. Additionally, stool sample kits are mailed to participants by the study team. The sample, returned directly to DayTwo, is labelled only with the participant ID number. DayTwo accounts are registered to participants under their ID number, and no other identifiers are shared.

## Abbreviations

ADA: American Diabetes Association
ASA24: Automated Self-Administered 24-Hour
AUC: Area Under the Curve
CDCES: Certified Diabetes Care and Education Specialist
CGM: Continuous Glucose Monitoring
CLIA: Clinical Laboratory Improvement Amendments
CONGA: Continuous Overall Net Glycemic Action
CV: Coefficient of Variation
DiaTeleMed: Diabetes Telemedicine Mediterranean Diet
EHR: Electronic Health Record
GLP1: Glucagon-Like Peptide 1
GV: Glycemic Variability
HbA1c: Hemoglobin A1c
HOMA2: Homeostatic Model Assessment 2
ICD: International Classification of Diseases
MAGE: Mean Amplitude of Glycemic Excursion
Mifflin St Jeor: Mifflin St Jeor Equation
NIH: National Institutes of Health
NYULH: New York University Langone Health
PNP: Personal Nutrition Project
PPGR: Postprandial Glycemic Response
RCT: Randomized Clinical Trial
RDN: Registered Dietitian Nutritionist
REDCap: Research Electronic Data Capture
SAS: Statistical Analysis Software
SCT: Social Cognitive Theory
SD: Standard Deviation
SGLT2: Sodium-Glucose Cotransporter-2
SPIRIT: Standard Protocol Items: Recommendations for Intervention Trials
STATA: STATA Software
T2D: Type 2 Diabetes
UCC: Usual Care Control
USDA: United States Department of Agriculture
WebEx: WebEx Videoconferencing

## References

[1] Centers for Disease Control and Prevention. National diabetes statistics report. https://www.cdc.gov/diabetes/data/statistics-report/index.html. Accessed 22 April 2024.

[2] ElSayed NA, Aleppo G, Aroda VR, Bannuru RR, Brown FM, Bruemmer D, 3. Prevention or delay of type 2 diabetes and associated comorbidities: standards of care in diabetes—2023. Diabetes Care. 2022;46(Supplement1):S41–8. 10.2337/dc23-S003 PMC9810464 36507633

[3] Writing Team for the Diabetes Control and Complications Trial/Epidemiology of Diabetes Interventions and Complications Research Group. Sustained effect of intensive treatment of type 1 diabetes mellitus on development and progression of diabetic nephropathy: the Epidemiology of Diabetes Interventions and Complications (EDIC) study. JAMA. 2003;290(16):2159–67. 14570951 10.1001/jama.290.16.2159 PMC2622725

[4] Holman RR, Paul SK, Bethel MA, Matthews DR, Neil HA. 10-year follow-up of intensive glucose control in type 2 diabetes. N Engl J Med. 2008;359(15):1577–89. 18784090 10.1056/NEJMoa0806470

[5] Gaede P, Lund-Andersen H, Parving HH, Pedersen O. Effect of a multifactorial intervention on mortality in type 2 diabetes. N Engl J Med. 2008;358(6):580–91. 18256393 10.1056/NEJMoa0706245

[6] Woerle HJ, Neumann C, Zschau S, Tenner S, Irsigler A, Schirra J, Impact of fasting and postprandial glycemia on overall glycemic control in type 2 diabetes Importance of postprandial glycemia to achieve target HbA1c levels. Diabetes Res Clin Pract. 2007;77(2):280–5. 17240473 10.1016/j.diabres.2006.11.011

[7] Monnier L, Colette C, Owens D. The glycemic triumvirate and diabetic complications: is the whole greater than the sum of its component parts? Diabetes Res Clin Pract. 2012;95(3):303–11. 22056719 10.1016/j.diabres.2011.10.014

[8] Evert AB, Dennison M, Gardner CD, Garvey WT, Lau KHK, MacLeod J, Nutrition therapy for adults with diabetes or prediabetes: a consensus report. Diabetes Care. 2019;42(5):731–54. 31000505 10.2337/dci19-0014 PMC7011201

[9] Heilbronn LK, Noakes M, Clifton PM. The effect of high- and low-glycemic index energy restricted diets on plasma lipid and glucose profiles in type 2 diabetic subjects with varying glycemic control. JACN. 2002;21(2):120–7. 10.1080/07315724.2002.10719204 11999539

[10] Wolever TM, Gibbs AL, Mehling C, Chiasson JL, Connelly PW, Josse RG, The Canadian Trial of Carbohydrates in Diabetes (CCD), a 1-y controlled trial of low-glycemic-index dietary carbohydrate in type 2 diabetes: no effect on glycated hemoglobin but reduction in C-reactive protein. Am J Clin Nutr. 2008;87(1):114–25. 18175744 10.1093/ajcn/87.1.114

[11] Ma Y, Olendzki BC, Merriam PA, Chiriboga DE, Culver AL, Li W, A randomized clinical trial comparing low-glycemic index versus ADA dietary education among individuals with type 2 diabetes. Nutrition. 2008;24(1):45–56. 18070658 10.1016/j.nut.2007.10.008 PMC2330083

[12] Sacks FM, Carey VJ, Anderson CA, Miller ER 3rd, Copeland T, Charleston J, Effects of high vs low glycemic index of dietary carbohydrate on cardiovascular disease risk factors and insulin sensitivity: the OmniCarb randomized clinical trial. JAMA. 2014;312(23):2531–41. 25514303 10.1001/jama.2014.16658 PMC4370345

[13] Reynolds AN, Tekinkaya H, Venn B. The effect on day-long glycemia of consuming lower and higher glycemic index diets in people with type 2 diabetes: a randomized crossover study. J Diabetes Metab. 2014;5:1–5.

[14] Jenkins DJ, Kendall CW, McKeown-Eyssen G, Josse RG, Silverberg J, Booth GL, Effect of a lowglycemic index or a high-cereal fiber diet on type 2 diabetes: a randomized trial. JAMA. 2008;300(23):2742–53. 19088352 10.1001/jama.2008.808

[15] Silva FM, Kramer CK, Crispim D, Azevedo MJ. A high-glycemic index, low-fiber breakfast affects the postprandial plasma glucose, insulin, and ghrelin responses of patients with type 2 diabetes in a randomized clinical trial. J Nutr. 2015;145(4):736–41. 25833777 10.3945/jn.114.195339

[16] Saslow LR, Kim S, Daubenmier JJ, Moskowitz JT, Phinney SD, Goldman V, A randomized pilot trial of a moderate carbohydrate diet compared to a very low carbohydrate diet in overweight or obese individuals with type 2 diabetes mellitus or prediabetes. PLoS ONE. 2014;9(4):e91027. 24717684 10.1371/journal.pone.0091027 PMC3981696

[17] Tay J, Luscombe-Marsh ND, Thompson CH, Noakes M, Buckley JD, Wittert GA, A very low-carbohydrate, low-saturated fat diet for type 2 diabetes management: a randomized trial. Diabetes Care. 2014;37(11):2909–18. 25071075 10.2337/dc14-0845

[18] Zeevi D, Korem T, Zmora N, Israeli D, Rothschild D, Weinberger A, Personalized nutrition by prediction of glycemic responses. Cell. 2015;163(5):1079–94. 26590418 10.1016/j.cell.2015.11.001

[19] Mendes-Soares H, Raveh-Sadka T, Azulay S, Edens K, Ben-Shlomo Y, Cohen Y, Assessment of a personalized approach to predicting postprandial glycemic responses to food among individuals without diabetes. JAMA Netw Open. 2019;2(2):e188102–e. 30735238 10.1001/jamanetworkopen.2018.8102 PMC6484621

[20] Berry SE, Valdes AM, Drew DA, Asnicar F, Mazidi M, Wolf J, Human postprandial responses to food and potential for precision nutrition. Nat Med. 2020;26(6):964–73. 32528151 10.1038/s41591-020-0934-0 PMC8265154

[21] NIH Nutrition Research Task Force. 2020–2030 Strategic plan for NIH nutrition research: a report of the NIH Nutrition Research Task Force Division of Program Coordination, Planning, and Strategic Initiatives (DPCPSI). https://dpcpsi.nih.gov/sites/default/files/2020NutritionStrategicPlan_508.pdf. Accessed 22 April 2024.

[22] Turnbaugh PJ, Ley RE, Mahowald MA, Magrini V, Mardis ER, Gordon JI. An obesity-associated gut microbiome with increased capacity for energy harvest. Nature. 2006;444(7122):1027–31. 17183312 10.1038/nature05414

[23] Mendes-Soares H, Raveh-Sadka T, Azulay S, Ben-Shlomo Y, Cohen Y, Ofek T, Model of personalized postprandial glycemic response to food developed for an Israeli cohort predicts responses in Midwestern American individuals. Am J Clin Nutr. 2019;110(1):63–75. 31095300 10.1093/ajcn/nqz028 PMC6599737

[24] Ben-Yacov O, Godneva A, Rein M, Shilo S, Kolobkov D, Koren N, Personalized postprandial glucose response-targeting diet versus mediterranean diet for glycemic control in prediabetes. Diabetes Care. 2021;44(9):1980–91. 34301736 10.2337/dc21-0162

[25] Rein M, Ben-Yacov O, Godneva A, Shilo S, Zmora N, Kolobkov D, Effects of personalized diets by prediction of glycemic responses on glycemic control and metabolic health in newly diagnosed T2DM: a randomized dietary intervention pilot trial. BMC Med. 2022;20(1):56. 35135549 10.1186/s12916-022-02254-y PMC8826661

[26] Intensive blood-glucose. control with sulphonylureas or insulin compared with conventional treatment and risk of complications in patients with type 2 diabetes (UKPDS 33). UK Prospective Diabetes Study (UKPDS) Group. Lancet. 1998;352(9131):837–53. 9742976

[27] Patel A, MacMahon S, Chalmers J, Neal B, Billot L, Woodward M, Intensive blood glucose control and vascular outcomes in patients with type 2 diabetes. N Engl J Med. 2008;358(24):2560–72. 18539916 10.1056/NEJMoa0802987

[28] Gerstein HC, Miller ME, Byington RP, Goff DC Jr., Bigger JT, Buse JB, Effects of intensive glucose lowering in type 2 diabetes. N Engl J Med. 2008;358(24):2545–59. 18539917 10.1056/NEJMoa0802743 PMC4551392

[29] Monnier L, Mas E, Ginet C, Michel F, Villon L, Cristol J-P, Activation of oxidative stress by acute glucose fluctuations compared with sustained chronic hyperglycemia in patients with type 2 diabetes. JAMA. 2006;295(14):1681–7. 16609090 10.1001/jama.295.14.1681

[30] Schisano B, Tripathi G, McGee K, McTernan PG, Ceriello A. Glucose oscillations, more than constant high glucose, induce p53 activation and a metabolic memory in human endothelial cells. Diabetologia. 2011;54(5):1219–26. 21287141 10.1007/s00125-011-2049-0

[31] Beck RW, Connor CG, Mullen DM, Wesley DM, Bergenstal RM. The fallacy of average: how using hba(1c) alone to assess glycemic control can be misleading. Diabetes Care. 2017;40(8):994–9. 28733374 10.2337/dc17-0636 PMC5521971

[32] Chan A-W, Tetzlaff JM, Gøtzsche PC, Altman DG, Mann H, Berlin JA, SPIRIT 2013 explanation and elaboration: guidance for protocols of clinical trials. BMJ. 2013;346:e7586. 23303884 10.1136/bmj.e7586 PMC3541470

[33] Hill NR, Oliver NS, Choudhary P, Levy JC, Hindmarsh P, Matthews DR. Normal reference range for mean tissue glucose and glycemic variability derived from continuous glucose monitoring for subjects without diabetes in different ethnic groups. Diabetes Technol Ther. 2011;13(9):921–8. 21714681 10.1089/dia.2010.0247 PMC3160264

[34] Wallace TM, Levy JC, Matthews DR. Use and abuse of HOMA modeling. Diabetes Care. 2004;27(6):1487–95. 15161807 10.2337/diacare.27.6.1487

[35] Battelino T, Danne T, Bergenstal RM, Amiel SA, Beck R, Biester T, Clinical targets for continuous glucose monitoring data interpretation: recommendations from the international consensus on time in range. Diabetes Care. 2019;42(8):1593–603. 31177185 10.2337/dci19-0028 PMC6973648

[36] Battelino T, Alexander CM, Amiel SA, Arreaza-Rubin G, Beck RW, Bergenstal RM, Continuous glucose monitoring and metrics for clinical trials: an international consensus statement. Lancet Diabetes Endocrinol. 2023;11(1):42–57. 36493795 10.1016/S2213-8587(22)00319-9

[37] Saboo B, Kesavadev J, Shankar A, Krishna MB, Sheth S, Patel V, Time-in-range as a target in type 2 diabetes: an urgent need. Heliyon. 2021;7(1):e05967. 33506132 10.1016/j.heliyon.2021.e05967 PMC7814148

[38] Barua S, Sabharwal A, Glantz N, Conneely C, Larez A, Bevier W, Dysglycemia in adults at risk for or living with non-insulin treated type 2 diabetes: insights from continuous glucose monitoring. EClinicalMedicine. 2021;35:100853. 33997745 10.1016/j.eclinm.2021.100853 PMC8093893

[39] Stanford Patient Education Research Center Self-Efficacy for Diabetes. https://www.slu.edu/medicine/family-medicine/pdfs/diabetes-management-selfefficacy-scale.pdf. Accessed 22 April 2024.

[40] Mifflin MD, St Jeor ST, Hill LA, Scott BJ, Daugherty SA, Koh YO. A new predictive equation for resting energy expenditure in healthy individuals. Am J Clin Nutr. 1990;51(2):241–7. 2305711 10.1093/ajcn/51.2.241

[41] ElSayed NA, Aleppo G, Aroda VR, Bannuru RR, Brown FM, Bruemmer D, 5. Facilitating positive health behaviors and well-being to improve health outcomes: standards of care in diabetes—2023. Diabetes Care. 2022;46(Supplement1):S68–96. 10.2337/dc23-S005 PMC9810478 36507648

[42] Bandura A. Social cognitive theory: an agentic perspective. Annu Rev Psychol. 2001;52:1–26. 11148297 10.1146/annurev.psych.52.1.1

[43] Bandura A. Self-efficacy: the exercise of control. New York: W.H. Freeman and Company; 1997.

[44] Tuncan SSUM, Mutlu HH, Oguz A. Evaluation of glycemic fluctuation as defined as the mean amplitude of glycemic excursion in hospitalized patients with type 2 diabetes. Cyprus J Med Sci 2016(1):37–41.

[45] Popp CJ, Hu L, Kharmats AY, Curran M, Berube L, Wang C, Effect of a personalized diet to reduce postprandial glycemic response vs a low-fat diet on weight loss in adults with abnormal glucose metabolism and obesity: a randomized clinical trial. JAMA Netw Open. 2022;5(9):e2233760. 36169954 10.1001/jamanetworkopen.2022.33760 PMC9520362

